# Down-regulation of circ_0058058 suppresses proliferation, angiogenesis and metastasis in multiple myeloma through miR-338-3p/ATG14 pathway

**DOI:** 10.1186/s13018-021-02867-8

**Published:** 2021-12-20

**Authors:** Lianguo Xue, Tao Jia, Yuanxin Zhu, Lidong Zhao, Jianping Mao

**Affiliations:** Department of Hematology, The First People’s Hospital of Lianyungang, No. 182 Tongguan North Road, Haizhou District, Lianyungang City, 222002 Jiangsu Province China

**Keywords:** circ_0058058, miR-338-3p, ATG14, Apoptosis, Metastasis, Multiple myeloma

## Abstract

**Background:**

Multiple myeloma (MM) is one of the most frequently diagnosed hematological malignancy. Dysregulation of circular RNAs (circRNAs) has important impacts on MM process. Herein, this work aimed to investigate the role and mechanism of circ_0058058 in MM progression.

**Methods:**

Levels of genes and proteins were detected by real-time reverse transcription PCR (RT-qPCR) and Western blot. CCK-8 assay, colony formation assay, EdU assay, flow cytometry, tube formation assay, transwell assay and Western blot were utilized to detect the proliferation, apoptosis, angiogenesis and metastasis of MM cells. The target relationship between miR-338-3p and circ_0058058 or ATG14 (autophagy related 14) was verified by dual-luciferase reporter assay and RNA immunoprecipitation (RIP) assay. In vivo experiments were performed using Xenograft assay.

**Results:**

Circ_0058058 was up-regulated in MM bone marrow aspirates and cells, knockdown of circ_0058058 reduced MM cell proliferation, angiogenesis and metastasis, but induced apoptosis in vitro. In a MM xenograft mouse model, circ_0058058 silencing reduced MM tumor growth and cell proliferation. Mechanistically, circ_0058058 acted as a sponge for miR-338-3p to up-regulate ATG14 expression, which was validated to be a target of miR-338-3p. Rescue assay showed that miR-338-3p inhibition reversed the antitumor effects of circ_0058058 knockdown on MM cell. Moreover, forced expression of miR-338-3p suppressed MM cell malignant phenotype, which was abolished by ATG14 up-regulation.

**Conclusion:**

Circ_0058058 functions as a sponge for miR-338-3p to elevate ATG14 expression to promote MM cell proliferation, metastasis and angiogenesis, affording a potential therapeutic target for MM prevention.

## Introduction

Multiple myeloma (MM) is second most frequently diagnosed hematological malignancy [1], characterized by high infiltration and accumulation of clonal malignant plasma cells in the bone marrow with initially asymptomatic [2, 3]. Although great advances in therapeutic strategies, including immunomodulatory drugs, proteasome inhibitors, and CD38 monoclonal antibody therapies, the survival rate of MM is still unsatisfactory with only about 1.5 years increase [4]. Therefore, an in-depth investigation on mechanisms underlying MM pathogenesis is of great essence for prolonging the survival of MM patients.

Circular RNAs (circRNAs) are a class of evolutionarily conserved non-coding RNAs formed by a covalently closed loop; thus, they can resist exonuclease-mediated degradation and are more stable than their linear isoforms [5, 6]. Moreover, circRNAs are more abundant, specific, conserved and highly organized in contrast to other RNA types [7]. Importantly, emerging evidence has showed the crucial role of circRNAs in regulating cellular biological processes linked with carcinogenesis, apoptosis, migration, and differentiation [8, 9]. Accordingly, circRNAs may be one of promising clinical biomarkers for different diseases. Recently, disturbance of circRNAs expression is implicated in the tumorigenesis of human malignancies [10, 11], including MM [12, 13]. Circ_0058058 is derived from its host gene ATIC (5-Aminoimidazole-4-Carboxamide Ribonucleotide Formyltransferase/IMP Cyclohydrolase), and it locates at chr2: 216177220-216190861 with the length of 512 bp. A recent study indicated that circ_0058058 increased EIF5A2 expression through miR-4319 to accelerate cell proliferation, invasion, and migration in hematological malignancy acute myeloid leukemia (AML) [14]. Moreover, it was also found to be up-regulated in MM patients [15]. Thus, we speculated that deregulation of circ_0058058 might be involved in the progression of MM.

Herein, this work investigated the potential role and mechanism of circ_0058058 in MM progression, which may provide a new avenue for the therapeutic intervention of MM.

## Materials and methods

### Specimen collection

Bone marrow aspirates were collected from 37 MM patients diagnosed based on the International Myeloma Working Group (IMWG) updated criteria at the First People’s Hospital of Lianyungang. A total of 37 age- and gender-matched healthy donors accepting bone marrow harvest for allogeneic transplantation were collected as normal controls. All tissues were immediately stored at -80℃ until used. All participants provided informed consent to obtain samples, and this work was authorized by the Ethics Committee of the First People’s Hospital of Lianyungang.

### Cell culture

Human MM cell lines (H929 and MM.1S) and normal bone marrow-derived plasma cells (nPCs) were purchased from American Type Culture Collection (ATCC; Manassas, VA, USA). MM cell line OPM2 was provided by Kyowa Hakko Kirin Co. Ltd. (Tokyo, Japan). Cell lines were cultured in 5% CO2 at 37 ℃ with RPMI-1640 medium (Life Technologies, Carlsbad, CA, USA) plus 10% fetal bovine serum (FBS (Life Technologies), 2 mmol/L L-glutamine, 100 U/mL of penicillin, and 100 μg/mL streptomycin (Solarbio, Shanghai, China).

### Real-time reverse transcription PCR (RT-qPCR)

The nuclear and cytoplasmic RNAs of H929 and MM.1S cells were obtained using the PARIS kit (Life Technologies). The isolation of total RNA was conducted using the Trizol reagent (Invitrogen, Carlsbad, CA, USA). The cDNAs were generated with the PrimeScript RT Reagent Kit (TaKaRa, Otsu, Japan) or miScript II RT Kit (Qiagen), and qPCR were carried out using SYBR Premix Ex Taq (TaKaRa). The molecular expression was calculated by the cycle threshold (Ct) value after normalization with reference control U6 or glyceraldehyde-3-phosphate dehydrogenase (GAPDH). The primer sequences were shown as follows:

- circ_0058058: F 5′-AGCCTTGAAGCCTTATTTAGTGT-3′, R 5′-TGGACTGCAGGATGCAAAGT-3′;
- ATIC: F 5′-CGGCCAGCTCGCCTTATTTA-3′, R 5′-CCAAGAGCGGTCAGGTTTCT-3′;
- ATG14 (autophagy related 14): F 5′-CATCATGGCGTCTCCCAGTG-3′, R 5′-CGATAAACCTCTCCCGGTCG-3′;
- miR-338-3p: F 5′-GCCGAGTCCAGCATCAGTGATT-3′, R 5′-CAGTGCAGGGTCCGAGGTAT-3′;
- GADPH: F 5′-CCCACATGGCCTCCAAGGAGTA-3′, R 5′-GTGTACATGGCAACTGTGAGGAGG-3′;
- U6: F 5′-CTCGCTTCGGCAGCACA-3′, R 5′-AACGCTTCACGAATTTGCGT-3′.

### Actinomycin D treatment

To block transcription, 5 μg/mL actinomycin D or dimethylsulfoxide (Solarbio) (negative control) was added into the culture medium of H929 and MM.1S cells, and the half-life of circ_0058058 and ATIC was determined by transcript levels at indicated time points with RT-qPCR.

### Cell transfection

The siRNAs for circ_0058058 (si-circ_0058058) and nontarget siRNA (si-NC), miR-338-3p mimic (miR-338-3p), inhibitor (anti-miR-338-3p) as well as negative control (miR-NC or anti-miR-NC) were synthesized by Genema (Shanghai, China). The full-length ATG14 was cloned into pcDNA 3.1 vector (Invitrogen) to overexpress ATG14 with empty pcDNA 3.1 as negative control (vector). Thereafter, the siRNAs, plasmids, miRNA mimic or inhibitor were transfected at concentrations of 100 nM, 100 ng or 40 nM, respectively, into H929 and MM.1S cells using Lipofectamine 2000 provided by Invitrogen.

For lentiviral clone transfection, lentivirus vectors carrying short hairpin RNA (shRNA) targeting circ_0058058 (sh-circ_0058058) or the control (sh-NC) were constructed by GenePharma Co., Ltd. (Shanghai, China). To obtain stable cell lines decreasing circ_0058058, H929 cells at 70% confluency were infected with recombinant lentiviral particles and polybrene (8 μg/mL) for 48 h. Stably expressing cells were identified using puromycin (2–5 μg/mL) for at least 1 week after infection, and the expression levels of circ_0058058 in stable clones were verified using RT-qPCR.

### Cell Counting Kit-8 (CCK-8) assay

Transfected H929 and MM.1S cells plated into 96-well plates were cultured at 37℃with 5% CO2 overnight. At 0, 24, 48, or 72 h incubation, each well was added with 10 µL CCK-8 reagent (Beyotime, Shanghai, China) and incubated at 37℃ for 2 h, and the absorbance at 450 nm was determined.

### Colony formation assay

After transfection, H929 and MM.1S cells at 1000 cells per well were plated into 6-well plates. Following 2 weeks culture, cells were fixed in 96% ethanol for 10 min, and cell colonies (> 50 cells) were imaged and analyzed after 0.5% crystal violet staining (Solarbio).

### Raj 5-Ethynyl-2'-deoxyuridine (EdU) assay

Transfected H929 and MM.1S cells were incubated with respective medium supplemented with 50 μM EdU labeling solution (RiboBio, Guangzhou, China) for 2 h. After being fixed with 4% paraformaldehyde and incubating with glycine, Apollo staining was carried out for 30 min. DNA staining was performed using 5 μg/mL DPAI for 30 min. EdU positive cells were detected by a fluorescence microscope.

### Flow cytometry

After 48 h of transfection, the apoptotic rates of H929 and MM.1S cells were determined by flow cytometry according to the manufacturer’s protocol of Annexin V-FITC/PI Apoptosis Kit (BD Biosciences, San Diego, CA, USA).

### Tube formation assay

The culture medium of H929 and MM.1S cells was replaced by serum-free RPMI-1640 medium for 48 h and then was collected, centrifuged and filtered to obtain tumor-conditioned medium (TCM). Each well of 96-well plates was coated with 50 μL Matrigel (BD Biosciences) for 30 min. HUVECs (1 × 10^5^) were starved in serum-free endothelial cell medium for 24 h and then placed at the gel with 200 mL TCM plus 1% FBS. The tube formation of HUVECs was imaged and analyzed using a microscope in the 12 h experimental period.

### Transwell assay

After transfection, H929 and MM.1S cells with serum-free medium were seeded into the upper chamber of transwell chambers (Costar, Corning, Switzerland) with polycarbonate films (for migration) or Matrigel-coated membrane (for invasion) (BD Biosciences), and 500 μL of complete culture medium plus 10% FBS was added into the bottom chamber. 24 h later, cells on the bottom surface of the membranes were counted manually with a microscope (Olympus) after staining with 0.1% crystal violet (Solarbio).

### Western blot assay

Equal amounts of protein isolated by using RIPA buffer (Beyotime) were loaded onto 10% sodium dodecyl sulfate polyacrylamide gel electrophoresis (SDS-PAGE) for separating, and then shifted onto polyvinylidene fluoride (PVDF) membranes (Millipore, Billerica, MA, USA). Then, the membranes were incubated with specific primary antibodies at 4℃overnight, including antibodies against E-cadherin (ab40772, 1:500), N-cadherin (ab18203, 1:1000), Vimentin (ab92547, 1:1000), ATG14 (ab80261, 1 μg/mL), and GAPDH (ab9485, 1:1000), followed by the interaction with corresponding secondary antibodies for 2 h at room temperature. The signal intensity was determined by the enhanced chemiluminescence system (Beyotime).

### Dual-luciferase reporter assay

Circ_0058058 or ATG14 3’UTR fragments covering wild-type (WT) target sites in miR-338-3p were amplified and inserted into the PGL3 Basic vector (Promega, Madison, WI, USA). Point mutations in binding sites of miR-338-3p were generated by Genema. Thereafter, each plasmid construct, and pRL-TK Renilla vector together miR-338-3p mimic or mimic negative control were co-transfected into H929 and MM.1S cells, and luciferase activities were examined after 48 h of transfection.

### RNA immunoprecipitation (RIP) assay

RIP assay was implemented according to the protocol of the EZMagna RIP Kit (Millipore). In brief, H929 and MM.1S cells were lysed by RIP lysis buffer, and cell lysates were incubated with IgG antibody or human Ago2 antibody conjugated magnetic beads. Finally, the co-precipitated RNAs were purified and subjected to RT-qPCR analysis.

### Mouse models

A murine model of human MM cells was established in female NOD.CB17-Prkdcscid/J mice ((*N* = 6/per group, 8-week-old, Charles River Labs, Beijing, China). H929 cells (1.2 × 10^7^) stably expressing lentiviral particles carrying sh-circ_0058058 or sh-NC were subcutaneously injected into mice to generate control and treatment groups. The tumor volume was measured every week. At day 28, mice were euthanized, and tumors were excised, weighed and fixed in formalin for IHC staining with Ki67 antibody as described previously [16] or collected for molecule detection with RT-qPCR and Western blot.

### Statistical analysis

All data were presented as the mean ± SD from at least three independent experiments and analyzed using the GraphPad Prism 6 software. The statistical differences were determined using a Student’s t test (two groups) or analysis of variance (more than two groups). *P* < 0.05 was defined as statistical significance.

## Results

### Circ_0058058 is up-regulated in MM bone marrow aspirates and cells

**Fig. 1:**
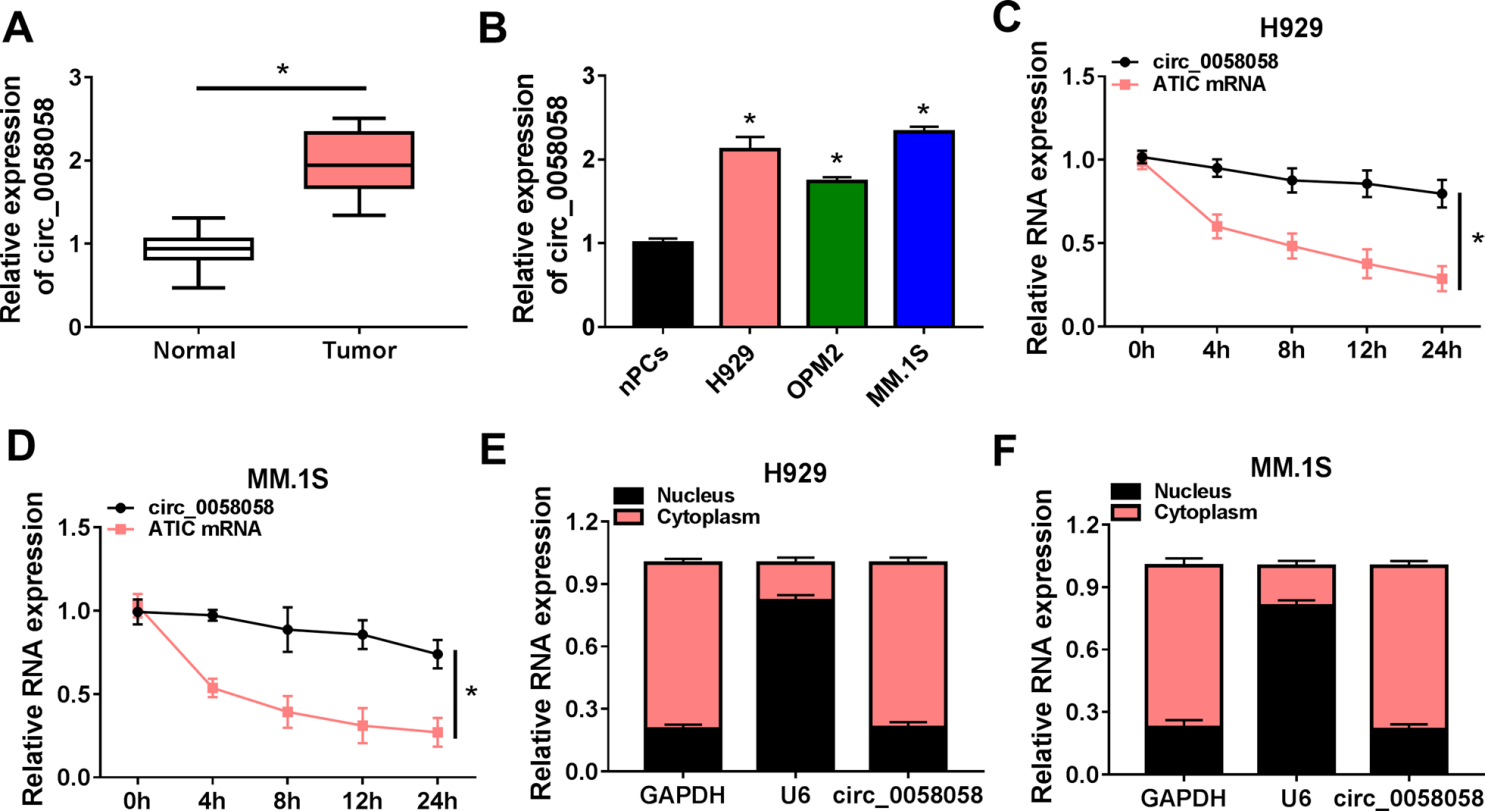
Circ_0058058 is up-regulated in MM bone marrow aspirates and cells. **A**, **B** RT-qPCR analysis of the expression level of circ_0058058 in bone marrow aspirates of MM patients and normal healthy donors, as well as in MM cell lines (H929, OPM2 and MM.1S) and normal nPCs cells. **C**, **D** RT-qPCR analysis of the relative RNA levels of circ_0058058 and ATIC in H929 and MM.1S cells after treatment with actinomycin D at the indicated time points. **E**, **F** The cellular distribution of circ_0058058 was analyzed by cellular RNA fractionation assays. *P < 0.05

To explore the role of circ_0058058 in MM progression, the expression profile of circ_0058058 was first investigated. As exhibited in Fig. 1A, circ_0058058 expression was higher in bone marrow aspirates of MM patients than those in normal control. Similarly, we also observed that circ_0058058 was highly expressed in MM cell lines (H929, OPM2 and MM.1S) compared with the nPCs cells (Fig. 1B). Then, the stability and localization of circ_0058058 in MM cells were investigated. Actinomycin D treatment assay suggested that the half-life of circ_0058058 exceeded 24 h, while that of ATIC mRNA was about 4 h in H929 and MM.1S cells (Fig. 1C, D), indicating that circ_0058058 was a stable circRNA. Besides that, it was also observed that circ_0058058 was predominately distributed in the cytoplasm of H929 and MM.1S cells (Fig. 1E, F). Therefore, these data suggested that circ_0058058 was a stable circRNA, and might be associated with the progression of MM.

### Knockdown of circ_0058058 suppresses MM cell proliferation, angiogenesis and metastasis in vitro

**Fig. 2:**
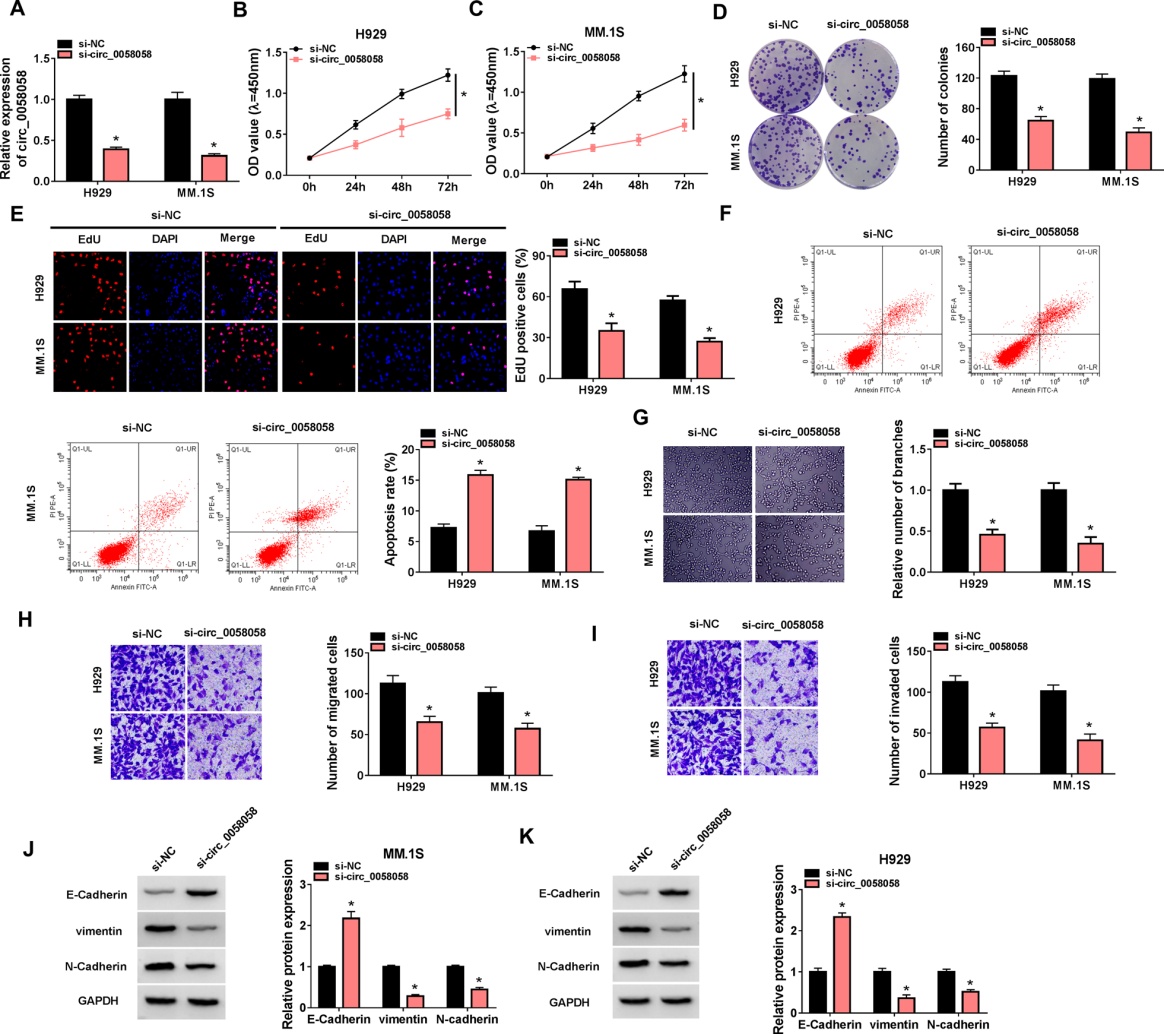
Knockdown of circ_0058058 suppresses MM cell proliferation, angiogenesis and metastasis in vitro*.* **A**–**K** H929 and MM.1S cells were transfected with si-circ_0058058 or si-NC. **A** RT-qPCR analysis of circ_0058058 expression in cells. Cell proliferation analysis using CCK-8 (**B**, **C**), colony formation (**D**), and EdU assays (**E**). **F** Flow cytometry for cell apoptosis. **G** Tube formation ability of HUVECs cultivated for 6 h in the TCM from transfected H929 and MM.1S cells. **H**, **I** Transwell assay for cell migration and invasion. **J**, **K** Western blot analysis of the protein levels of E-cadherin, vimentin and N-cadherin. *P < 0.05

Next, the detailed functions of circ_0058058 in MM cell malignant phenotype were investigated. We knocked down circ_0058058 by using specific siRNAs in H929 and MM.1S cells, and found that the introduction of si-circ_0058058 significantly reduced circ_0058058 expression in MM cell (Fig. 2A). Functionally, circ_0058058 down-regulation led to a decrease in cell proliferation in H929 and MM.1S cells, evidenced by the inhibition of cell proliferation rate (Fig. 2B, C), colony-forming activity (Fig. 2D) and DNA synthesis activity (Fig. 2E). Conversely, circ_0058058 silencing induced apoptosis in H929 and MM.1S cells (Fig. 2F). Besides, decreased tube formation was observed with HUVECs that were cultivated in the TCM from circ_0058058-down-regulated H929 and MM.1S cells relative to those that grew in the TCM from si-NC cells (Fig. 2G). Additionally, transwell assay showed that circ_0058058 silencing reduced the migration and invasion abilities of H929 and MM.1S cells (Fig. 2H, I). Moreover, Western blot analysis suggested that the protein levels of vimentin and N-cadherin were decreased, and E-cadherin protein level was increased in circ_0058058-decreased H929 and MM.1S cells (Fig. 2J, K), indicating the inhibition of EMT process. Taken together, knockdown of circ_0058058 suppressed MM cell malignant phenotype in vitro.

### MiR-338-3p is a target of circ_0058058

**Fig. 3:**
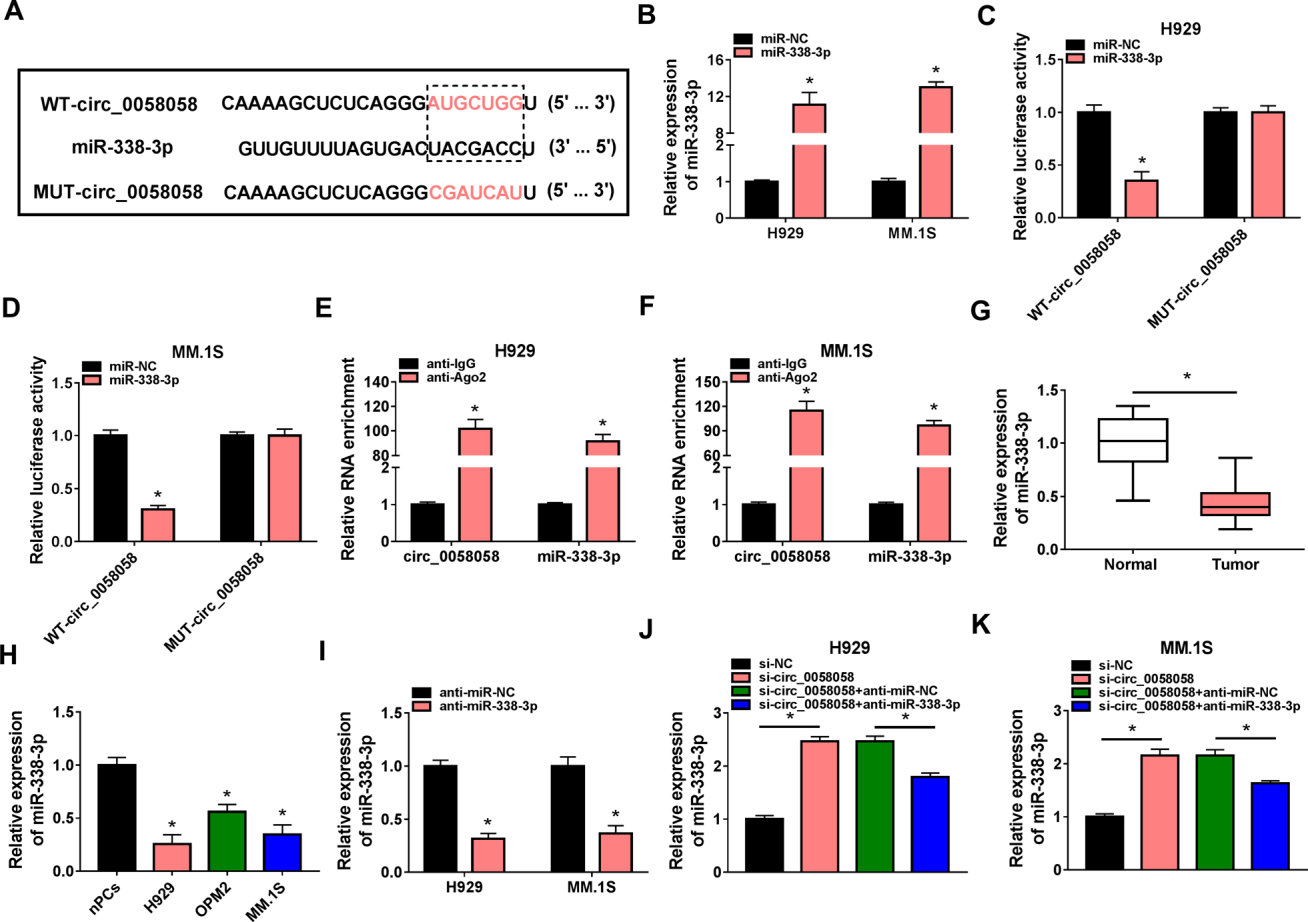
MiR-338-3p is a target of circ_0058058. **A** The putative binding site between circ_0058058 and miR-338-3p. **B** RT-qPCR analysis of miR-338-3p expression in cells transfected with miR-338-3p mimic or miR-NC. **C**, **D** Dual-luciferase reporter assay for the luciferase activity of wild and mutated circ_0058058 reporter after miR-338-3p overexpression in H929 and MM.1S cells. **E**, **F** Anti-Ago2 RIP assay was used in H929 and MM.1S cells to determine circ_0058058 and miR-338-3p RNA enrichment in immunoprecipitate complexes. **G**, **H** RT-qPCR analysis of miR-338-3p expression level in bone marrow aspirates of MM patients and normal healthy donors, as well as in MM cell lines (H929, OPM2 and MM.1S) and normal nPCs cells. **I** Transfection efficiencies of miR-338-3p inhibitor or inhibitor NC. **J**, **K** RT-qPCR analysis of miR-338-3p expression level in H929 and MM.1S cells transfected with si-NC, si-circ_0058058, si-circ_0058058 + anti-miR-NC, or si-circ_0058058 + anti-miR-338-3p

Given that circ_0058058 was mainly distributed in the cytoplasm of H929 and MM.1S cells, we assumed that circ_0058058 might probably regulate gene expression at the posttranscriptional level by sponging microRNAs (miRNAs). Therefore, the downstream miRNAs of circ_0058058 were explored. Bioinformatics analysis using Circular RNA Interactome database revealed that circ_0058058 contained a putative binding site of miR-338-3p (Fig. 3A). After confirming the elevation efficiency of miR-338-3p mimic or mimic NC using RT-qPCR (Fig. 3B), we performed dual-luciferase reporter assay. The results showed that miR-338-3p mimic overtly reduced the luciferase activity of wile-type circ_0058058 reporter vector but not the mutated reporter vector in H929 and MM.1S cells (Fig. 3C, D). Moreover, RIP assay confirmed that miR-338-3p and circ_0058058 were preferentially pulled down by anti-Ago2 pellet relative to the IgG immunoprecipitates in H929 and MM.1S cells (Fig. 3E, F), further verifying the binding between miR-338-3p and circ_0058058. Thereafter, the expression pattern of miR-338-3p in MM was investigated. Compared to healthy controls, miR-338-3p was found to be decreased in MM patients (Fig. 3G). Also, a decrease in miR-338-3p expression in MM cells was observed compared with nPCs cells (Fig. 3H). The inhibitor of miR-338-3p was confirmed to decreased miR-338-3p expression significantly compared with inhibitor NC (Fig. 3I). Furthermore, it was observed that circ_0058058 knockdown caused an increase in miR-338-3p expression, which was reduced by miR-338-3p inhibitor in H929 and MM.1S cells (Fig. 3J, K). Therefore, we confirmed that circ_0058058 targeted miR-338-3p and negatively regulated its expression.

### Circ_0058058 regulates proliferation, angiogenesis and metastasis in MM cells via miR-338-3p

**Fig. 4:**
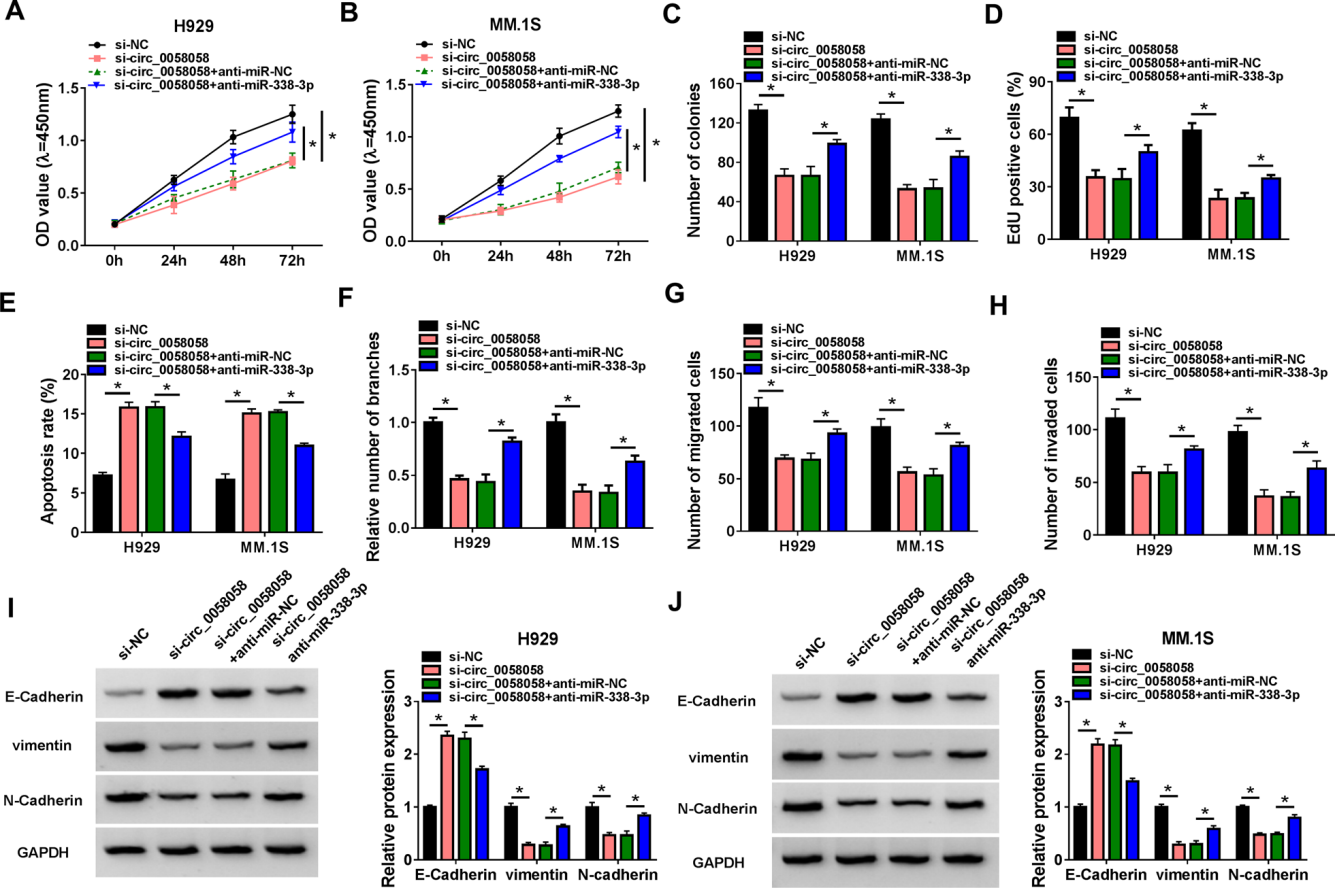
Circ_0058058 regulates proliferation, angiogenesis and metastasis in MM cells via miR-338-3p. **A**–**J** H929 and MM.1S cells were transfected with si-NC, si-circ_0058058, si-circ_0058058 + anti-miR-NC, or si-circ_0058058 + anti-miR-338-3p. Cell proliferation analysis using CCK-8 (**A**, **B**), colony formation (**C**), and EdU assays (**D**). **E** Flow cytometry for cell apoptosis. **F** Tube formation ability of HUVECs cultivated for 6 h in the TCM from transfected H929 and MM.1S cells. **G**, **H** Transwell assay for cell migration and invasion. **I**, **J** Western blot analysis of the protein levels of E-cadherin, vimentin and N-cadherin. *P < 0.05

To assess whether the effects of circ_0058058 on MM cell phenotype were mediated by miR-338-3p, we performed rescue assay by co-transfection of si-circ_0058058 and anti-miR-338-3p into H929 and MM.1S cells. Results from CCK-8, colony formation, EdU assays and flow cytometry manifested that miR-338-3p inhibition reversed circ_0058058 knockdown-induced suppression of H929 and MM.1S cells proliferation (Fig. 4A–D) and enhancement of cell apoptosis (Fig. 4E). Moreover, down-regulation of miR-338-3p suppressed the inhibitory effects of circ_0058058 silencing on tube formation (Fig. 4F), migration (Fig. 4G), and invasion (Fig. 4H) in H929 and MM.1S cells. Besides that, miR-338-3p inhibition promoted EMT process in circ_0058058-decreased H929 and MM.1S cells by increasing vimentin and N-cadherin protein level and decreasing E-cadherin protein level (Fig. 4I, J). Altogether, circ_0058058/miR-338-3p axis was responsible for the progression of MM cell malignant phenotype.

### ATG14 is a target of miR-338-3p

**Fig. 5:**
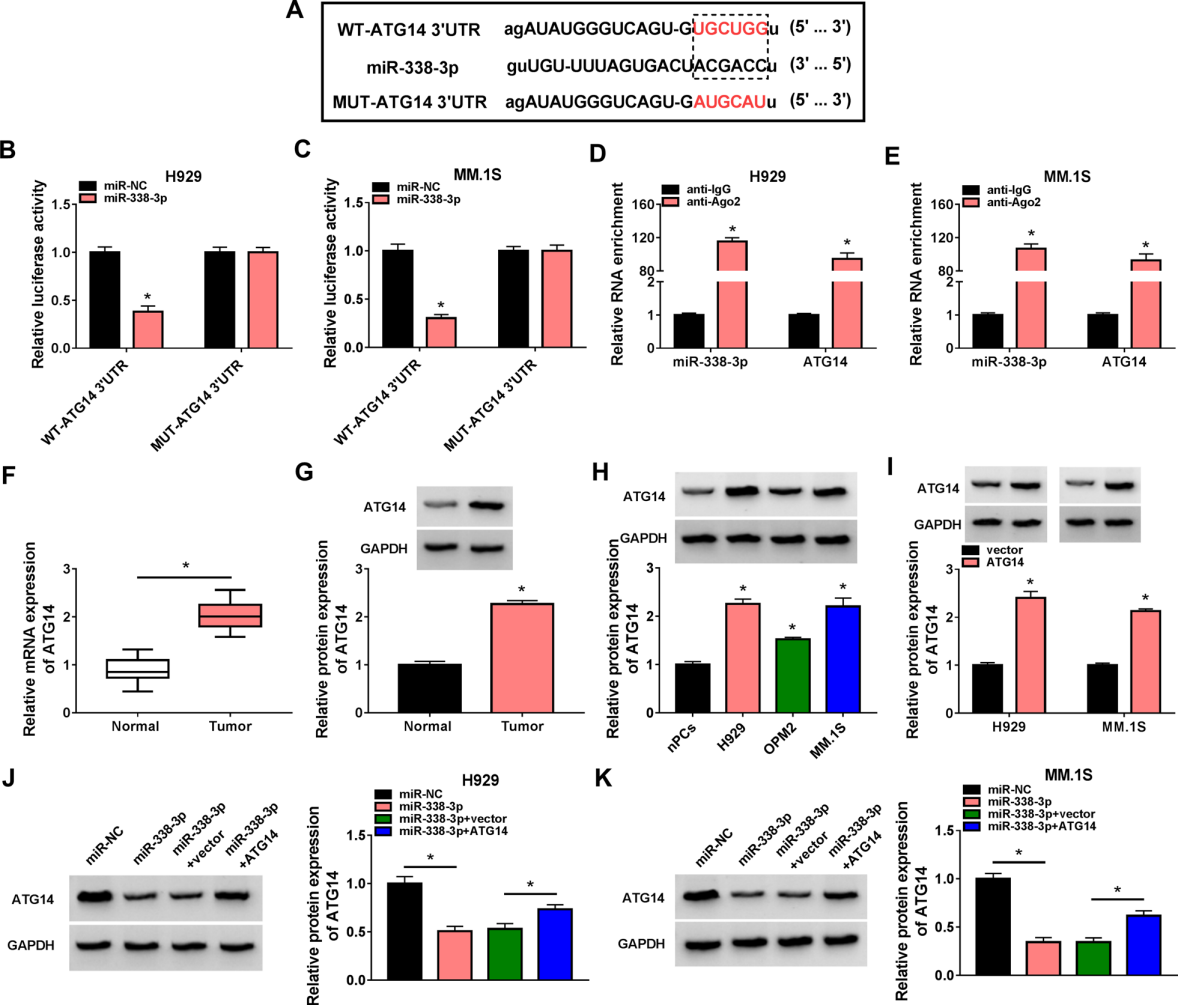
ATG14 is a target of miR-338-3p. **A** The putative binding site between ATG14 and miR-338-3p. **B**, **C** Dual-luciferase reporter assay for the luciferase activity of wild and mutated ATG14 reporter after miR-338-3p overexpression in H929 and MM.1S cells. **D**, **E** Anti-Ago2 RIP assay was used in H929 and MM.1S cells to determine ATG14 and miR-338-3p RNA enrichment in immunoprecipitate complexes. **F**, **G** RT-qPCR and Western blot analysis of ATG14 expression level in bone marrow aspirates of MM patients and normal healthy donors. **H** Western blot analysis of ATG14 expression level in MM cell lines (H929, OPM2 and MM.1S) and normal nPCs cells. **I** Western blot analysis of ATG14 protein level in H929 and MM.1S cells transfected with ATG14 or vector. **J**, **K** Western blot analysis of ATG14 protein level in H929 and MM.1S cells transfected with miR-NC, miR-338-3p, miR-338-3p + vector, or miR-338-3p + ATG14. *P < 0.05

The downstream genes of miR-338-3p were then explored. According to the prediction of starBase database, miR-338-3p contained a putative binding site of ATG14 (Fig. 5A). The results of dual-luciferase reporter assay revealed that co-transfection of miR-338-3p mimic and wile-type ATG14 reporter vector led to a decrease in the luciferase activity in H929 and MM.1S cells, while there was no change in cells co-transfection with miR-338-3p mimic and mutated ATG14 reporter vector (Fig. 5B, C). Moreover, RIP assay confirmed that miR-338-3p and ATG14 were preferentially pulled down by anti-Ago2 pellet compared to the IgG immunoprecipitates in H929 and MM.1S cells (Fig. 5D, E). All these data verified the binding between miR-338-3p and ATG14. The expression of ATG14 was discovered to be increased in MM patients both at mRNA and protein levels (Fig. 5F, G); moreover, an increase in ATG14 expression in MM cells was also obtained by Western blot (Fig. 5H). Additionally, after validation of the transfection efficiency of ATG14 overexpression vector, it was proved that miR-338-3p mimic reduced ATG14 expression in H929 and MM.1S cells (Fig. 5I), while this condition was rescued by ATG14 vector introduction (Fig. 5J, K). Thus, we confirmed that miR-338-3p targetedly suppressed ATG14 expression.

### MiR-338-3p suppresses MM cell proliferation, angiogenesis and metastasis via ATG14

**Fig. 6:**
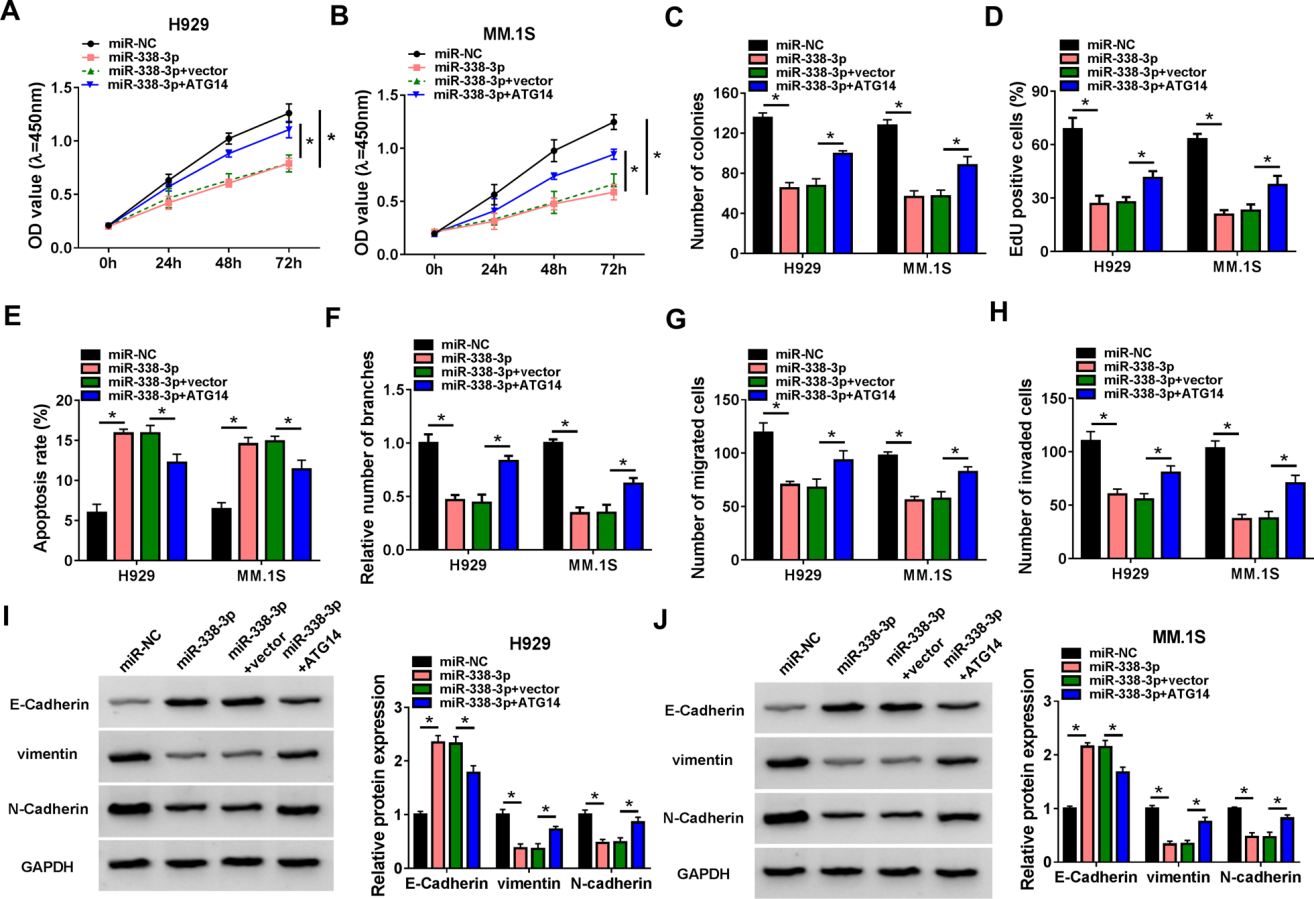
MiR-338-3p suppresses MM cell proliferation, angiogenesis and metastasis via ATG14. **A**–**J** H929 and MM.1S cells were transfected with miR-NC, miR-338-3p, miR-338-3p + vector, or miR-338-3p + ATG14. Cell proliferation analysis using CCK-8 (**A**, **B**), colony formation (**C**), and EdU assays (**D**). **E** Flow cytometry for cell apoptosis. **F** Tube formation ability of HUVECs cultivated for 6 h in the TCM from transfected H929 and MM.1S cells. **G**, **H** Transwell assay for cell migration and invasion. **I**, **J** Western blot analysis of the protein levels of E-cadherin, vimentin and N-cadherin. *P < 0.05

To evaluate the role of miR-338-3p/ATG14 axis in MM cell malignant phenotype, H929 and MM.1S cells co-transfected with miR-338-3p mimic and ATG14 vector were used to conduct rescue assay. Functionally, miR-338-3p overexpression suppressed cell proliferation (Fig. 6A–D) and induced cell apoptosis (Fig. 6E) in H929 and MM.1S cells, which were attenuated by ATG14 up-regulation. Besides, miR-338-3p overexpression suppressed tube formation of H929 and MM.1S cells, while this condition was reversed by ATG14 increase (Fig. 6F). Moreover, both the suppression of cell migration and invasion mediated by miR-338-3p overexpression were abolished by ATG14 up-regulation (Fig. 6G, H). Furthermore, ATG14 increase also reduced miR-338-3p overexpression-evoked arrest of EMT process in H929 and MM.1S cells (Fig. 6I, J). Collectively, miR-338-3p/ATG14 axis was engaged in the regulating of MM cell malignant phenotype.

### Circ_0058058 acts as a sponge for miR-338-3p to regulate ATG14 expression

**Fig. 7:**
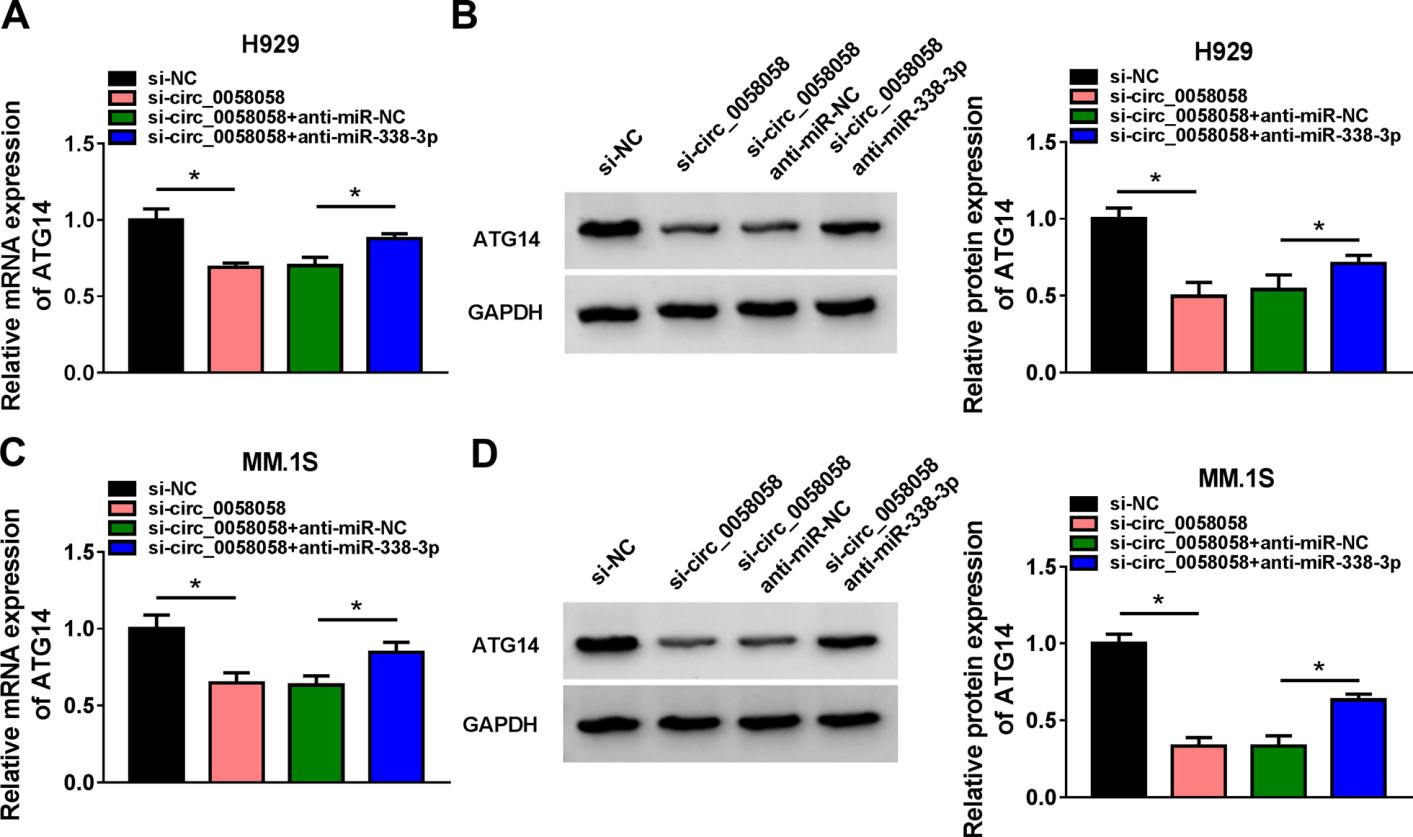
Circ_0058058 acts as a sponge for miR-338-3p to regulate ATG14 expression. **A**–**D** RT-qPCR and Western blot analysis of ATG14 expression level in H929 and MM.1S cells transfected with si-NC, si-circ_0058058, si-circ_0058058 + anti-miR-NC, or si-circ_0058058 + anti-miR-338-3p. *P < 0.05

Subsequently, the influence of circ_0058058/miR-338-3p axis on ATG14 expression was investigated. As shown in Fig. 7A–D, circ_0058058 knockdown led to a significant reduction of ATG14 expression both at mRNA and protein levels, which were rescued by the inhibition of miR-338-3p in H929 and MM.1S cells, suggesting the circ_0058058/miR-338-3p/ATG14 feedback loop in MM cells.

### Knockdown of circ_0058058 inhibits MM growth in xenograft murine models

**Fig. 8:**
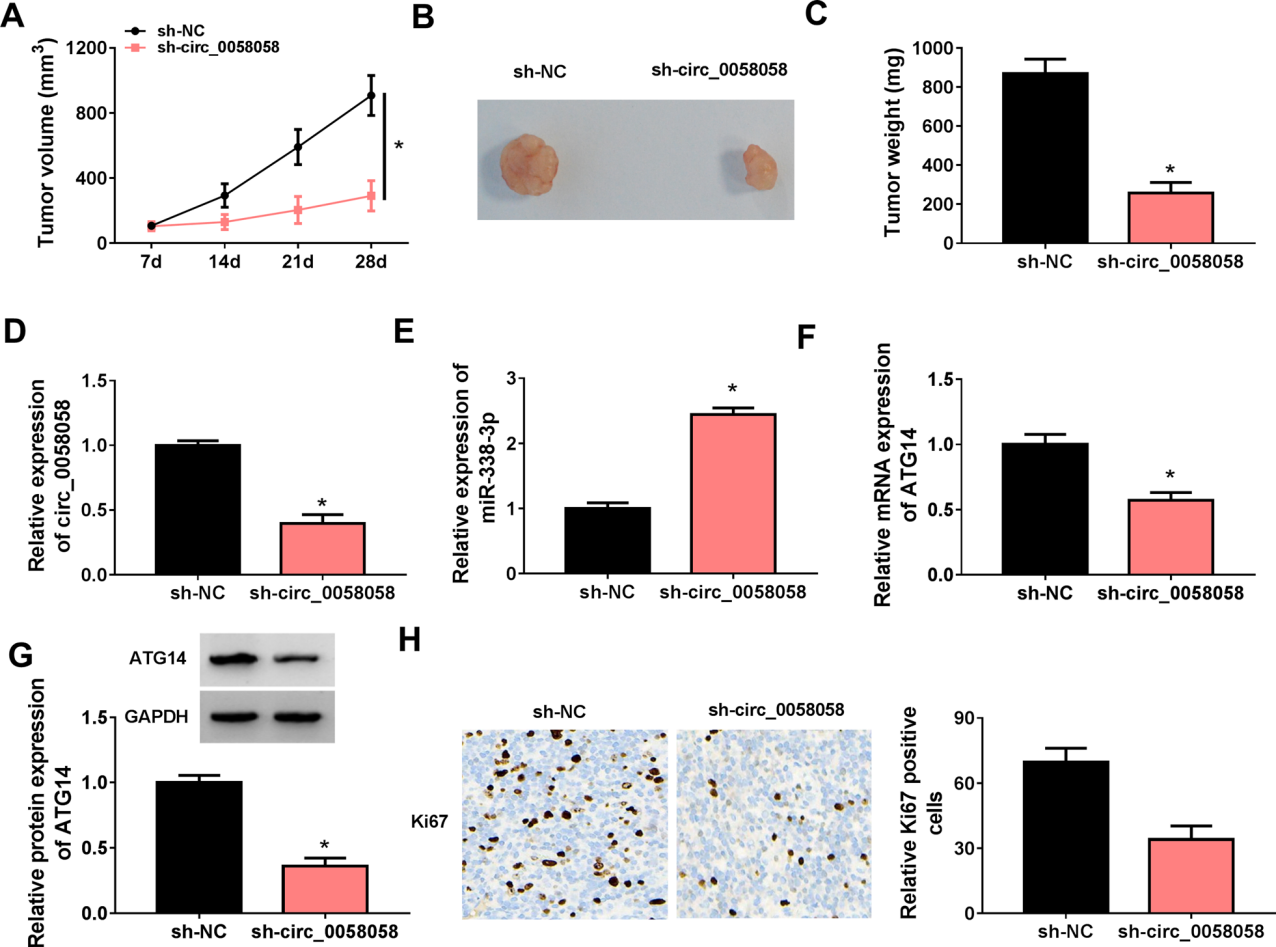
Knockdown of circ_0058058 inhibits MM growth in xenograft murine models. **A** The volume of MM tumors was detected every week after treatment for 7 days. **B** Typical MM tumors after subcutaneous injection of H929 cells stably knockdown circ_0058058. **C** The weight of MM tumors was detected after treatment for 28 days. **D**–**G** RT-qPCR and Western blot analysis of circ_0058058, miR-338-3p and ATG14 expression in xenograft of each group. **H** IHC staining for ki67 protein level in xenograft of each group. *P < 0.05

To validate our in vitro findings that circ_0058058 knockdown could restrain MM, we examined its ability to inhibit tumor growth in xenograft murine models. Xenograft growth of MM was strikingly suppressed by circ_0058058 knockdown compared with the control animals (Fig. 8A–C). Treatment of H929 cells with sh-circ_0058058 led to the decrease in circ_0058058 and ATG14 expression levels and an elevation of miR-338-3p expression level in xenograft tissues (Fig. 8D–G). Moreover, the protein level of Ki67 was significantly decreased in xenograft tissues of sh-circ_0058058 group compared with sh-NC group (Fig. 8H). In all, these results validated the anticancer effect of circ_0058058 silencing in MM in vivo.

## Discussion

MM is a unique B-cell neoplasm, accounting for 10% of all hematological malignancies [17]. Owing to the disseminates of plasma cells into multiple bone lesions, MM patients often show a poor prognosis [18]. Thus, a good understanding of the molecular mechanism underlying the pathogenesis of MM could thus advance the development of therapeutic strategies for MM patients.

CircRNAs are a type of abundant, highly conserved, stable, and tissue-specific regulatory RNAs, which have been revealed to play significant effects on a variety of biological processes; moreover, circRNAs dysregulation is closely associated with the tumorigenesis and treatment response of hematological malignancies [19]. In MM, some circRNAs have also been reported to participate in this malignancy progression. For instance, circ_0000190 was demonstrated to repress MM tumorigenesis and growth in vitro and in MM model mice [20]. Liu et al*.* suggested that circITCH overexpression sensitized drug resistant MM cells to bortezomib, thus reducing MM tumorigenesis [21]. Besides that, circ-CDYL was revealed to act as an oncogene to contribute to the uncontrolled growth of MM [22]. Therefore, targeting circRNAs in MM will be an attractive treatment strategy. In current research, we observed that circ_0058058 expression was higher in bone marrow aspirates of MM patients and cell lines. Functionally, knockdown of circ_0058058 led to the reduction of cell proliferation, tube formation, migration, invasion abilities, inhibition of EMT process and acceleration of cell apoptosis in vitro. Moreover, the therapeutic potential of administering circ_0058058 antagonist in mice-bearing H929 cells subcutaneous tumors was also validated that circ_0058058 silencing hindered the growth of MM tumor in vivo. Thus, circ_0058058 functions an oncogene in the progression of MM.

CircRNAs distributed in the cytoplasm can bind to certain miRNAs by acting as miRNA sponges to regulate gene transcription and translation [23, 24]. Owing to the distribution of circ_0058058 in the cytoplasm of MM cells, the target miRNAs of circ_0058058 were investigated. MiRNAs have been revealed that can modulate various cellular functions by posttranscriptionally regulating target genes, and altered miRNA expression using overexpressing plasmids or small interfering RNAs has clinical potential for the treatment of diseases [25–27]. MiR-338-3p is considered as tumor suppressor, which was demonstrated to suppress cancer progression in many types of malignancies, such as colorectal cancer, prostate cancer, breast cancer and so on [28–30]. Moreover, recent reports displayed that miR-338-3p was decreased in MM patients, miR-338-3p restoration suppressed MM cell mobility and growth [31, 32]. In this study, we also observed a decrease in miR-338-3p expression in MM patients and cells; furthermore, its overexpression reduced MM cell growth, tube formation and metastasis. Importantly, we confirmed that circ_0058058 targeted miR-338-3p, and further rescue assay showed that miR-338-3p silencing attenuated the suppressive action of circ_0058058 knockdown on MM cell malignant phenotypes.

In the present work, we also validated that miR-338-3p directly targeted ATG14; moreover, it was also observed that circ_0058058 could regulate ATG14 expression through binding to miR-338-3p. ATG14 is an essential autophagy-specific regulator, which has a critical role in cellular senescence and autophagy [33]. In MM, ATG14 was discovered to be up-regulated, knockdown of ATG14 reduced cell proliferation and induced apoptosis, thus sensitizing myeloma cells to melphalan [34]. In this work, an increase in ATG14 level was also obtained in MM patients and cells; moreover, ATG14 up-regulation abolished the anticancer functions of miR-338-3p on MM tumorigenesis.

## Conclusion

**Fig. 9:**
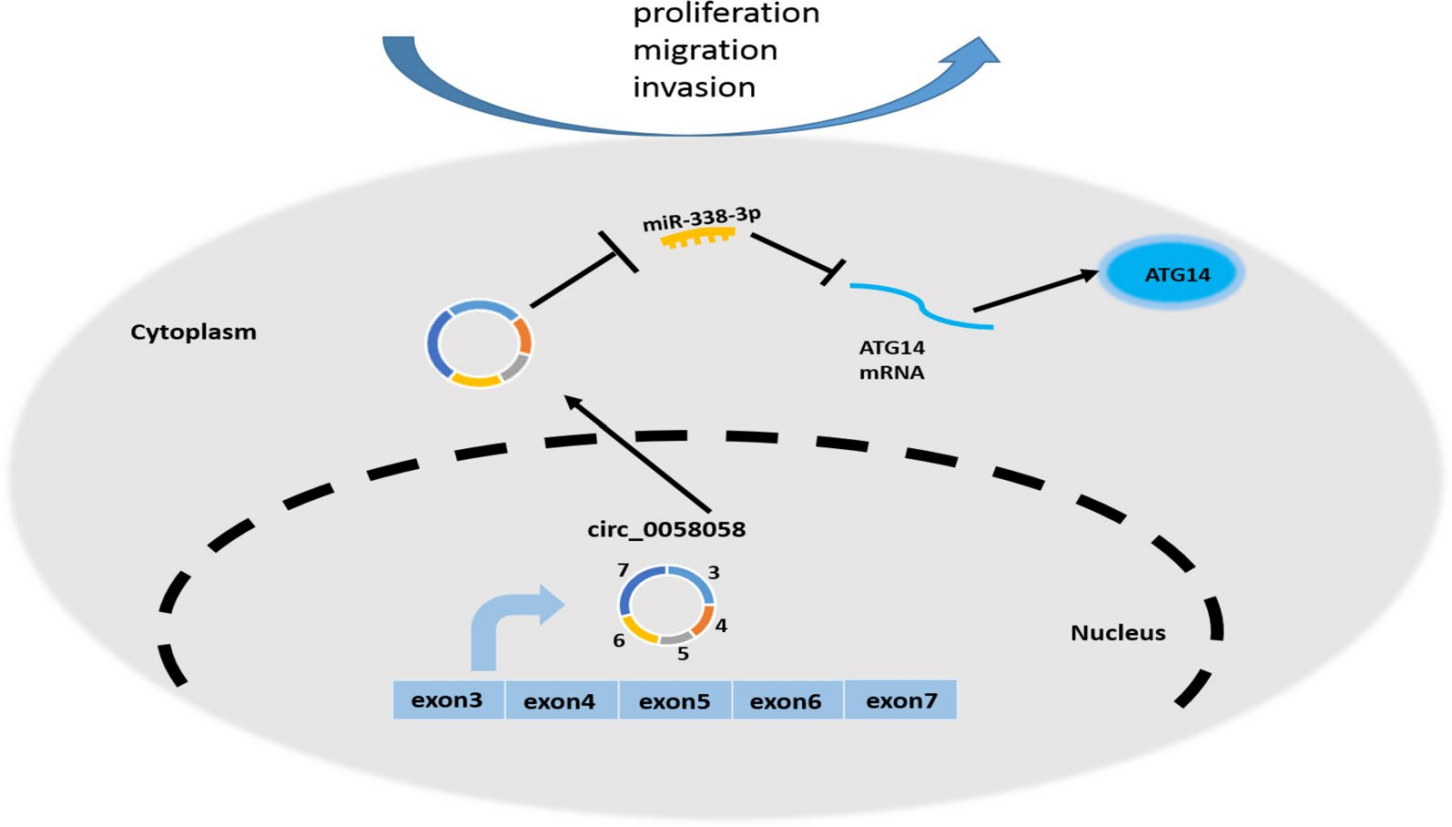
Schematic diagram of circ_0058058/miR-338-3p/ATG14 feedback loop in MM progression. Circ_0058058 up-regulates ATG14 through sponging miR-338-3p to promote MM cell proliferation and metastasis

In conclusion, this study first validated that circ_0058058 up-regulated ATG14 expression through miR-338-3p to promote MM cell growth and metastasis (Fig. 9), suggesting a novel therapeutic target for MM treatment.

## Data Availability

Not applicable.

## Abbreviations

MM: Multiple myeloma
circRNAs: Circular RNAs
RT-qPCR: Real-time reverse transcription PCR
RIP: RNA immunoprecipitation
